# Illinois State Medical Society

**Published:** 1870-05

**Authors:** 


ILLINOIS STATE MEDICAL SOCIETY, March 22, 1870.
*Office of the Permanent Secretary,* 181 *West Madison St., Chicago, Ill.*

The twentieth annual session of this society will be held in Dixon, Lee county, on the third Tuesday in May, 1870, at 10 A. M. The following Committees are expected to report: On the Practice of Medicine, Dr. C. Goodbrake, of Clinton, Chairman; on Surgery, Dr. Moses Gunn, of Chicago, Chairman; on Obstetrics, Dr. Elias Wenger, of Gillman, Chairman; on Drugs and Medicines, Dr. Charles Hunt, of Dixon, Chairman; on Necrology, Dr. J. H. Hollister, of Chicago, Chairman.

Special Committees:—On Criminal Abortions, Dr. DeLaskie Miller, of Chicago, Chairman; on Ophthalmology, Dr. J. S. Hildreth, of Chicago, Chairman; on Pulmonary Tuberculosis, Dr. J. P. Ross, of Chicago, Chairman; on the use of Plaster Paris in Fractures, Dr. R. G. Bogue, of Chicago; on Otology, Dr. Sam'l J. Jones, of Chicago, Chairman; on Arrangements, Drs. Everett, Hunt, Phillips, of Dixon, Dr. J. C. Corbus, of Amboy, and Dr. E. P. Cook, of Mendota; on Medical Education, Dr. N. S. Davis, of Chicago, Chairman.

Secretaries of all medical organizations are requested to send lists of their delegates to the Permanent Secretary as soon as elected. Medical societies are entitled to one delegate for every five members. Any respectable physician unable to attend as a delegate, or permanent member, may attend as a member by invitation, on the recommendation of the Committee of Arrangements.

T. D. FITCH.

### *Note to the Editor.*

[The following note should have been earlier printed. We think Dr. Ames was the first *matriculant* of the College.—Ed.]

Chicago, March 12, 1870.

Prof. J. Adams Allen, M.D., Ed. Chicago Med. Journal—*Doctor:* On reading, in the March number of the Journal, Dr. Ames' address before the alumni of Rush College, I observe a foot-note states: "Dr. Ames was the first graduate of the College." A just regard for the truth of history compels me to correct the error contained in the note referred to. Dr. Ames was *not* the first graduate of Rush College; the undersigned was the first graduate of Rush Medical College. Trusting that a correction of this error will be noted in the Journal, I am, respectfully,

Your ob't servant,

Wm. Butterfield, M. D.

### *Brainard Medical Society.*

The Society met in the Court House, in Winamac, Indiana, April 7th, 1870. Dr. J. W. C. Eaton, read a paper on "Some of the Uses and Actions of Ipecacuanha." Dr. Hoag reported a case of Epilepsia. Dr. Washburn reported a case of Uterine Hæmorrhage, caused by partial detachment of the placenta, complicated with rigidity of the os. Drs. Hoag and Cleland, reported, each, a case of shoulder presentation.

The fourth annual election of officers resulted as follows: J. W. C. Eaton, *President;* I. B. Washburn, *Secretary;* Wm. Kelsey, *Treasurer;* F. B. Thomas, J. B. Hoag, W. T. Cleland, *Censors.*

I. B. WASHBURN, *Sec'y.*

